# 

**Published:** 1882-10

**Authors:** 


					﻿^VOL-IL,
■OCTOBER, 1882.
NO. 10.
THE
HOMEOPATHIC pHY^lClAfl,
A MONTHLY JOURNAL OF MEDICAL SCIENCE.
J “ JZa<zn« est verilas, el proevalebit.” ■
IE. CT. LEK LK. ID.
EDITOR.
CONl'ENTS:
(See inside of Cover.)
PHILADELPHIA:.
No. 2109 CHESTNUT STREET.
T. O nsr x> OJET.
ALFRED/HEATH & CO., Sole Agents fob Great Britain,
. 114 Ebury Street, Eaton Square.
Yearly Subscription, $2.00, invariably in advance.
) Entered at the Philadelphia Post-Office, as Second-Class Mail Matter.
				

